# Clustering-Triggered Emission of EPS-605 Nanoparticles and Their Application in Biosensing

**DOI:** 10.3390/polym14194050

**Published:** 2022-09-27

**Authors:** Chengcheng Li, Xiaotong Shi, Xiaodong Zhang

**Affiliations:** 1 College of Light Industry and Food Engineering, Utilization of Forest Resources, International Innovation Center for Forest Chemicals and Materials, Jiangsu Co-Innovation Center for Efficient Processing, Nanjing Forestry University, Nanjing 210037, China; 2 Division of Chemistry and Biological Chemistry, School of Physical and Mathematical Sciences, Nanyang Technological University, Singapore 637371, Singapore

**Keywords:** exopolysaccharides, polymeric nanoparticles, clustering-triggered emission, Fe^3+^ detection, nonconventional luminogens, *Lactiplantibacillus plantarum*

## Abstract

Natural carbohydrates with intrinsic luminescent properties have drawn increasing attention thanks to their fundamental importance and promising applications. To expand the range of natural nonconventional biomacromolecule luminogens and to gain deep insights into their emission mechanism, we prepared EPS-605, a naturally occurring spherical nanoparticle based on negatively charged exopolysaccharides (EPS), and studied its emission behavior. It was found that EPS-605 was highly emissive in the aggregate state, such as powder and film. Furthermore, EPS-605 aqueous solutions exhibited concentration-enhanced emission characteristics. According to fluorescence spectra and confocal images, the fluorescence phenomenon of EPS-605 was not affected by the pH value and the carbon sources. The emission behavior of EPS-605 was attributed to the clustering-triggered emission (CTE) mechanism. Moreover, EPS-605 was successfully utilized for Fe^3+^ detection since its fluorescence could be selectively quenched by Fe^3+^. It could be used to detect Fe^3+^ with a low limit of detection (0.06 μM) and a wide detection range from 0.05 to 250 μM. Overall, these findings not only benefit the exploitation of EPS-based nonconventional biomacromolecule luminogens, but also reveal the potential applications of EPS-605 in biosensing/bioimaging, anticounterfeiting, and encryption owing to its excellent biocompatibility, environmental friendliness, and intrinsic photoluminescence property.

## 1. Introduction

Luminescence phenomena play important roles in our modern technology and daily life [1]. Fluorescent organic luminogens or nanoparticles have attracted considerable attention due to their unique photophysical properties and extensive applications in various fields such as bioimaging, biosensing, drug delivery, and optoelectronics [2,3,4]. However, traditional fluorescent organic luminogens have shown inherent drawbacks, such as high cytotoxicity, low photostability, nonbiodegradability, poor water solubility, complex synthesis process, and aggregation-caused quenching (ACQ) effect, limiting their practical applications [4,5,6]. In 2001, Tang and co-workers found that pentaphenylsilole is virtually nonluminescent in solution, but highly emissive in the aggregate state [7], which is antithetic to the ACQ phenomenon, when the concept of aggregation-induced emission (AIE) was coined for this phenomenon [8]. Compared with ACQ materials, AIE materials have multiple advantages including excellent photostability and high fluorescence intensity in the aggregate state, thus making them suitable for long-time and wash-free imaging with a high signal-to-noise ratio [9].

Different from the conventional conjugated luminogens with classic chromophores serving as emitting units, some polymers containing flexible chains and tunable structures without conventional chromophores can also emit fluorescence under certain conditions [1,10]. These nonconventional luminophores can exhibit intriguing intrinsic luminescence, including AIE, cluster-triggered emission (CTE), concentration-enhanced emission, excitation-dependent luminescence, and prevailing phosphorescence [11]. Compared with conventional conjugated light-emitting polymers, the nonconventional luminogens have the advantages of simple preparation, eco-friendliness, outstanding biocompatibility, and high hydrophilicity [12], rendering their comprehensive promising application in biomedical areas and optoelectronic devices [13,14]. More importantly, such nonconventional luminogens satisfy the need of emitting fluorescence at a high concentration or in a solid state for some applications [15]. This luminescence phenomenon has been found in various dendritic or hyperbranched compounds rich in nitrogen (N), oxygen (O), and sulfur (S), such as poly(amidoamine), polyvinylpyrrolidone, polyethylenimine, and poly(amido acid) [16,17]. Some compounds rich in ester and carbonyl groups can also exert photoluminescence, including PMV [17], aliphatic oximes [14], and nonaromatic polyurethanes [3]. Moreover, some natural products can also emit fluorescence or phosphorescence in a solid or concentrated state, such as sodium alginate [18], rice [19], starch [20], cellulose [21], and protein [22]. Instead of aromatics contributing to the fluorescence of the conventional conjugated luminogens, they are generally equipped with some traditional auxochromic groups [11]. As an increasing number of unconventional polymers are found to be able to exert photoluminescence under specific conditions, various luminescence mechanisms have been proposed, including oxidation of tertiary amines, aggregation of C=O groups, electron cloud overlap [12], oxidation, architecture effect, terminal group effect, and pH influence [23,24]. Despite this, the light-emitting mechanism of such polymers is still controversial, driving the development of novel polymers for a deeper understanding of their emission mechanism. Recently, Dou et al. reported that sodium alginate (SA) showed CTE and persistent room temperature phosphorescence [12]. Wang et. al. investigated the emissive mechanism of the nonaromatic compound (xylitol) and polymers (poly(ethylene glycol) and pluronic F127), exhibiting their CTE property [25]. This mechanism has also been adapted to nonconjugated polyacrylonitrile [26], such that the CTE phenomenon is observed in synthetic compounds and natural biomolecules [12,26,27,28].

On the other hand, metal ions may cause serious health problems for humans and affect the ecosystem and environment [29,30,31], while others play important roles in the life system. Among them, Fe^3+^ is an important metal ion in the human body and plays an essential role in a series of biological processes, such as the biosynthesis of enzymes involved in oxygen uptake, oxygen metabolism, and electron transfer [32,33]. The turbulence of Fe^3+^ levels will severely affect normal life activities. Thus, detecting Fe^3+^ is particularly necessary to ensure the regular circulation of Fe^3+^ in the human body. Nowadays, conventional metal detection strategies include spectrophotometry, atomic absorption spectrometry, and inductively coupled plasma mass spectrometry [34]. However, there are some drawbacks with these traditional methods, such as long detection time, complicated analysis procedure, poor selectivity, a large stationary instrument, high test cost, etc. [35]. In comparison, fluorescence spectroscopy is a sensitive detection method with low cost, facile operation, and good reproducibility, and thus has been well developed to apply in metal ion detection [22,36]. For example, the fluorescent nitrogen-doped carbon quantum dots were synthesized for monitoring Hg^2+^ and Ag^+^ ions with detection limits of 4.8 and 1 nM, respectively [37]. Fluorescence probes can interact with various metals and lead to a change in the fluorescence intensity, which can be exploited as a fluorescent sensor. However, these fluorescent probes usually contain poisonous and expensive metal semiconductor materials or suffer from laborious synthetic routines. Thus, there is an urgent need to develop low-cost and eco-friendly alternative materials. The application of environmentally friendly, sustainable, and safe strategies or reagents to detect heavy metals has been of great interest [34].

Exopolysaccharides (EPSs), which are derived from microorganisms, are high-molecular-weight carbohydrate polymers with enormous structural diversity and structural controllability [22]. EPSs are generally composed of monosaccharides and noncarbohydrate substituents such as acetate, phosphate, pyruvate, and succinate [38]. They are divided into two types according to whether they are composed of the same monosaccharide, namely homopolysaccharides and heteropolysaccharides [38]. EPSs play essential roles in biological systems and possess enormous potential applications in bioremediation, biomedicine, cosmetics, the food industry, and wastewater treatment due to their cost-effective, biodegradable, safe, accessible, reusable, and environmentally friendly properties [22]. The function of EPSs is not only related to their chemical compositions and conformation, but also closely related to their physical−chemical properties [39]. Some natural polysaccharides have been reported to be able to emit fluorescence, including sodium alginate [12], starch [40], cellulose [40], and carboxymethylated nanocellulose [28]. Our previous study reported a type of EPS produced by *Lactobacillus plantarum* (*L. plantarum*) LCC-605 that was isolated from traditional Chinese pickles in Yunnan province [30]. The EPS could be self-assembled into nanoparticles in water with a diameter of ~88 nm, which was used as an effective biosorbent of heavy metals and organic dyes owing to their abundant functional groups (including -OH, -COOH, and -NH_2_) and large specific surface area [30]. They have also been used as a drug delivery system for photodynamic therapy [41]. Herein, the luminescence properties of the naturally occurring EPS-605 nanoparticles were investigated to extend their functionality and applications. We found that dilute solutions of EPS-605 are virtually nonluminescent, but aggregation or high concentrations make them emissive. The emission behavior of EPS-605 was also elucidated. Furthermore, a representative application using the EPS-605 NPs in Fe^3+^ ion detection was investigated. The present work may open a new frontier for expanding the type of luminescent biomacromolecules and the understanding of their emission mechanism.

## 2. Materials and Methods

### 2.1. Materials

Strain *L. plantarum* LCC-605 was preserved in our lab [30]. Dialysis bags with the molecular weight cut-off (MWCO) of 14 kDa were from Spectrum Labs (Rancho Dominguez, USA). CaCl_2_, CuNO_3_∙3H_2_O, FeSO_4_, MgCl_2_, PbCl_2_, MnSO_4_∙7H_2_O, AlCl_3_∙6H_2_O, FeCl_3_∙6H_2_O, CoCl_2_∙6H_2_O, AgNO_3_, ZnCl_2_, NaCl, and CdCl_2_ were purchased from Sigma-Aldrich. Other chemicals used in this study were of analytical grade.

### 2.2. Preparation of EPS-605 Solution, Powder, and Film

EPS-605 was prepared as described in our previous study [30]. EPS-605 powder was obtained by freeze-drying the extracted EPS solutions, with EPS-605 film prepared using the method reported by Dou et al. [12]. Briefly, the stock solution was prepared by dissolving 40 mg of EPS-605 powder in 7.0 mL of ultrapure water that was subsequently stirred for 2 h at 80 °C. After removing the air bubbles by ultrasound, the stock solution was cast into polyethylene dishes and dried at 25 °C to evaporate most of the water to obtain the EPS film.

### 2.3. Characterization

Photographs of EPS-605 powder, film, and solutions were taken under white light or the irradiation of 302 nm in a Gel imager (Tanon-2500, Shanghai, China). The UV–vis absorption spectra of EPS-605 solutions were obtained with a Shimadzu UV-2600 spectrophotometer. The fluorescence intensities of the samples at different concentrations (0, 0.05, 0.1, 0.2, 0.4, 0.8, 1.0, 2.0, and 5.0 mg/mL) were then measured on a fluorescence spectrometer (PerkinElmer, Waltham, MA, USA). Fluorescent lifetimes (τ) of different EPS-605 materials were measured on an ultrafast time-resolved fluorescence lifetime spectrometer (Life Spec II, Edinburgh Instruments, Livingston, UK). Quantum yields (Φ) were measured on a PL quantum yield measurement system (Hamamatsu Photonics, C9920-02G, Hamamatsu, Japan) with an excitation wavelength (λex) of 320 nm.

### 2.4. Confocal Observation

For the confocal imaging of the EPS-605, EPS-605 powder obtained from different carbon sources was loaded onto a glass slide and covered with a cover glass, which was secured with tape and observed using an inverted confocal laser scanning microscope (CLSM) TCS SP8 (Leica, Wetzlar, Germany) with a 100× oil immersion objective. The excitation wavelength was 488 nm and the fluorescence emission was detected in the wavelength range of 582–670 nm.

### 2.5. Ion Detection

The ion solutions, including Ca^2+^, Cu^2+^, Fe^2+^, Mg^2+^, Pb^2+^, Mn^2+^, Al^3+^, Fe^3+^, Co^2+^, Ag^+^, Zn^2+^, Na^+^, and Cd^2+^ at a concentration of 200 μM, were separately prepared in pure water. Then, 10 μL of the above metal ions was added to 5 mL of EPS-605 aqueous solutions (0.8 mg/mL), respectively, and stayed for 10 min. The fluorescence intensities of EPS-605 aqueous solutions containing different ions were then determined using a fluorescence spectrometer (PerkinElmer, Waltham, MA, USA). The different emission intensities at 450 nm were recorded upon 302 nm excitation. The luminescence photographs of EPS-605 containing different ions under 302 nm UV light were also taken.

Fourier transform infrared (FTIR) spectra were collected using an FTIR spectrometer (VERTEX 80V; Bruker, Billerica, MA, USA). Peak fit software v4.12 was used to smooth and fit the spectra.

## 3. Results

### 3.1. Fluorescence Property of EPS-605

**Figure 1:**
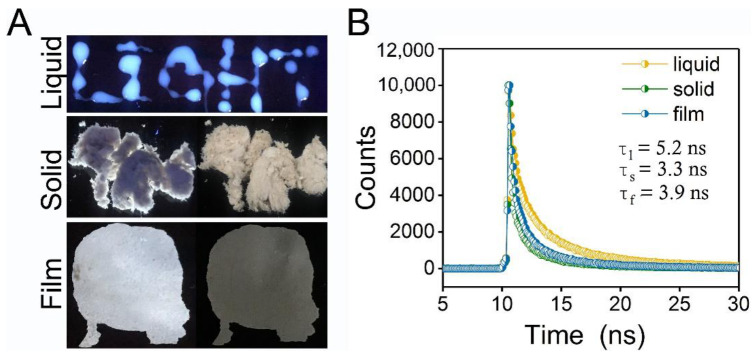
(**A**) Photographs of EPS-605 aqueous solutions (at the concentration of 0.8 mg/mL by writing the word “LIGHT”), powder, and film taken under 302 nm (left) or white light condition (right). (**B**) Time-resolved fluorescence decay curves of EPS-605 aqueous solutions, solid powder, and film monitored at 450 nm (λex = 302 nm).

Some natural polymers are intrinsically luminescent and can exert emissive properties, attributed to CTE or AIE mechanisms. In our previous study, we obtained a heteropolysaccharide from *L. plantarum* that could be self-assembled into spherical nanoparticles in water with a diameter of ~88 nm, named EPS-605 [30]. These self-assembled nanoparticles were clustered in water observed by SEM, exhibiting excellent adsorption capabilities for dyes and heavy metal ions [30]. More interestingly, we found that EPS-605 possessed emissive property with bright blue–white emissions in an aqueous solution when excited at 302 nm, which was evidenced by writing the word “LIGHT” using the solution at a concentration of 0.8 mg/mL (Figure 1A). To have a better understanding of the basic fluorescence characteristic and to pave the application of EPS-605, the luminescence properties of EPS-605 in different states were investigated. Figure 1A shows the luminescence photographs of EPS-605 in aqueous solution, solid powder, and film under 302 nm UV light. EPS-605 emitted fluorescence (left) when excited with a 302 nm UV lamp in powder and film states (Figure 1A). A similar phenomenon was also observed in rice with starch as the main component triggered by the AIE mechanism reported by Tang and coworkers [19,20]. The quantum yield (QY) of EPS-605 in film, powder, and liquid states was 1.5%, 1.6%, and 2.6%, respectively. The fluorescence lifetime of EPS-605 aqueous solution (0.8 mg/mL) was determined to be 5.2 ns, which was longer than that in solid powder (3.3 ns) and film (3.9 ns) states. As far as we know, this is the first time that the fluorescence of heteropolysaccharides from *L. plantarum* has been observed. Our findings extend the ranges and types of natural intrinsically luminescent polymers and the functionality of EPS.

### 3.2. Concentration-Enhanced Emission of EPS-605

**Figure 2:**
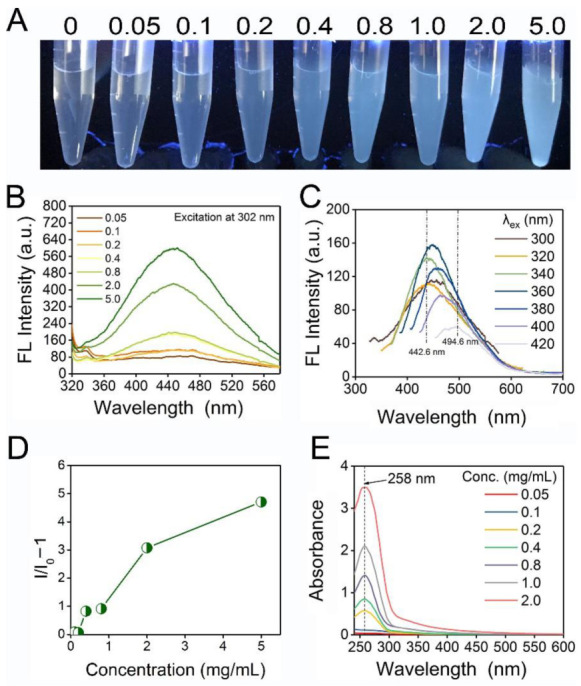
(**A**) Photographs of EPS-605 aqueous solutions at different concentrations under 302 nm light irradiation. (**B**) FL emission spectra of EPS-605 at different concentrations (0.05–5.0 mg/mL) excited at 302 nm. (**C**) FL emission spectra of EPS-605 aqueous solutions at a fixed concentration of 0.8 mg/mL under different excitations. (**D**) Fluorescence intensity changes at 442.6 nm (I/I_0_ −1) of EPS-605 as a function of its concentration. (**E**) Absorption spectra of EPS-605 aqueous solutions at different concentrations.

To have a better understanding of the photophysical properties of EPS-605, different concentrations (0.05–5.0 mg/mL) of EPS-605 were investigated for its luminescence property. It was nonemissive in dilute solutions (below 0.2 mg/mL) with extremely low fluorescence signals almost parallel to the control (Figure 2A,B). Weak luminescence signals were observed when the concentration of EPS-605 reached 0.2 mg/mL, at which a rather faint but visible emission was observed with an emission peak located at ~451.8 nm under 302 nm excitation (Figure 2B). Remarkably, enhanced emission was observed as the concentration of EPS-605 increased to 0.4 mg/mL (Figure 2B). Notably, for concentrated solutions (i.e., 0.8–5 mg/mL), bright blue–white emissions from EPS-605 were observed upon 302 nm UV excitation. Similar to the fluorescence properties of sodium alginate reported by Dou [12], EPS-605 also exhibited concentration-enhanced emission properties, rather than the aggregation-caused quenching (ACQ) effect [42]. Meanwhile, the emission maxima of EPS-605 varied from 442.6 to 494.6 nm while its excitation wavelength changed from 300 to 420 nm (Figure 2C), displaying its excitation-dependent emission. The excitation-dependent emission might be ascribed to the presence of a heterogeneous population of emissive species. Furthermore, to investigate the characteristic of EPS-605, the absorbance of EPS-605 with different concentrations was recorded using a UV-vis spectrometer. As shown in Figure 2D,E, the dilute aqueous solutions of EPS-605 (below 0.2 mg/mL) showed negligible absorptions parallel to the baseline, whereas the absorption of concentrated solutions (above 0.2 mg/mL) was progressively enhanced with increasing concentration, accompanied by the appearance of a peak at ~258 nm (Figure 2E). Moreover, the Mie effect and obvious Tyndall effect (Figure S1) in concentrated solutions with a concentration equal to and above 0.2 mg/mL caused a significant deviation from the baseline, which is consistent with the above results.

### 3.3. Influence of Carbon Source and pH on the Fluorescence Property of EPS-605

**Figure 3:**
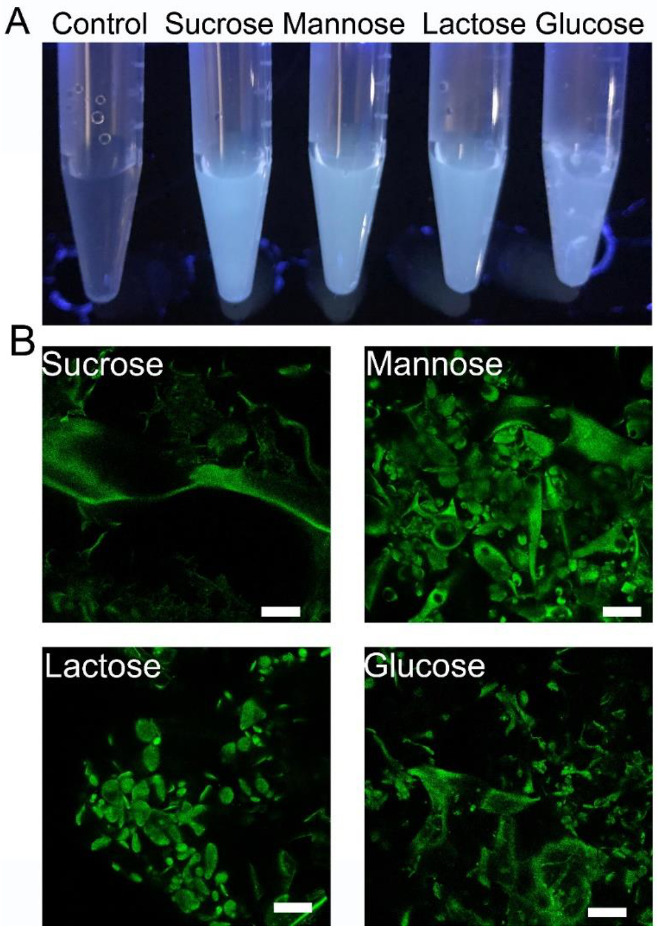
(**A**) Photographs of EPS-605 aqueous solutions obtained from different carbon sources taken under 302 nm UV light. (**B**) Confocal images of EPS-605 powder produced from sucrose, mannose, lactose, and glucose carbon sources excited at 488 nm. (Scale bar = 25 μm).

To further elucidate the fluorescence characteristics of EPS-605, the emission property of EPS-605 produced under different carbon sources (sucrose, mannose, lactose, and glucose) were compared. As exhibited in Figure 3A, all EPS-605 solutions produced from different carbon sources could emit fluorescence. According to the fluorescence intensity, EPS-605-sucrose was the strongest, followed by EPS-605-mannose, EPS-605-lactose, and EPS-605-glucose. The confocal images in Figure 3B also proved that the fluorescence phenomenon was not affected by the carbon sources for culturing of *L. plantarum* LCC-605. We also studied the influence of pH on the fluorescent intensity using EPS-605-glucose as a representative. The negligible influence of pH on the fluorescence property of EPS-605 solutions was observed (Figure S2). Such intrinsic emission behavior of EPS-605 may endow great potential in bioimaging, chemo-sensing, encryption, etc. [3,12]. Nevertheless, the luminescence mechanism of EPS-605 needs to be further explored in the future.

### 3.4. Response to Metal Ions by EPS-605

**Figure 4:**
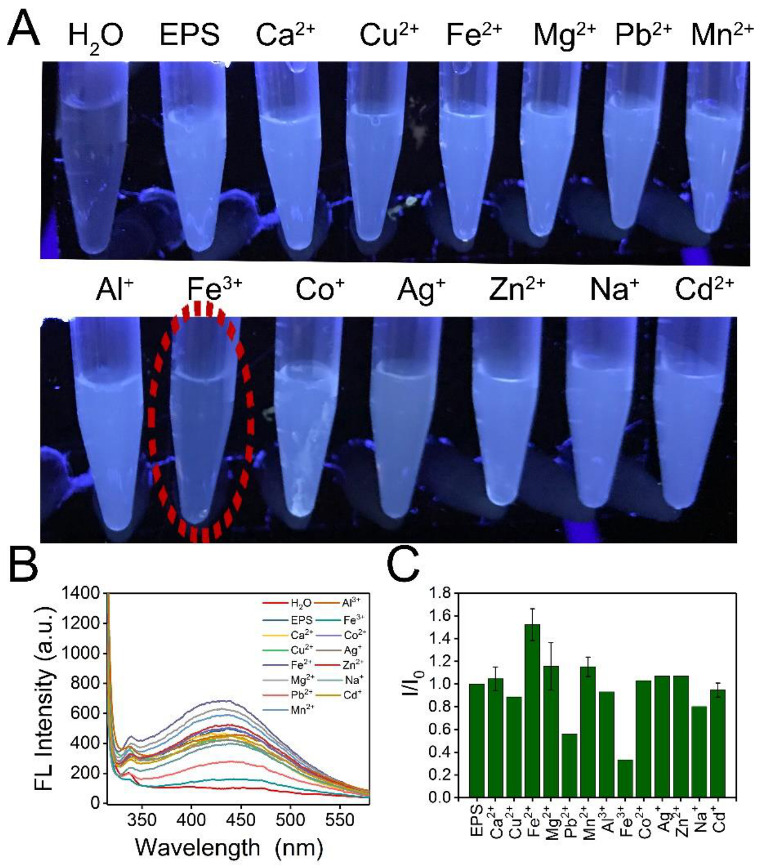
(**A**) Photographs of EPS solutions (0.8 mg/mL) in the presence or absence of various metal ions (200 μM) taken under 302 nm UV light (The red dashed circle represented the fluorescence quenching of EPS-605 after adding Fe^3+^). (**B**) Fluorescence spectra of EPS-605 incubated with various metal ions (200 μM) in an aqueous solution under 302 nm excitation. (**C**) Changes of fluorescence intensity (I/I_0_) at 302 nm versus various metal ions.

The fluorescence responses of EPS-605 aqueous solutions (0.8 mg/mL) as a result of the addition of other cations at a concentration of 200 μM, including Ca^2+^, Cu^2+^, Fe^2+^, Mg^2+^, Pb^2+^, Mn^2+^, Al^3+^, Fe^3+^, Co^2+^, Ag^+^, Zn^2+^, Na^+^, and Cd^2+^, were investigated. Photographs, fluorescence spectra, and fluorescence intensity changes of EPS-605 solutions in the presence or absence of various metal irons were displayed in Figure 4. The fluorescence intensities of EPS-605 decreased dramatically due to the introduction of Fe^3+^ compared with the addition of other metal ions. Fe^3+^ reduced the fluorescence intensity of EPS-605 by approximately 70%. These results suggest that EPS-605 has superior selectivity towards Fe^3+^ than other metal ions, which might be attributed to the present carboxyl/hydroxyl groups on the surface of EPS-605 that can interact with Fe^3+^ [30]. Based on this observation, EPS-605 could be used as a fluorescent probe to detect Fe^3+^. Compared with the metal-ion-containing fluorescent probes, the EPS-605 probe is much safer and more friendly to the environment and human health.

### 3.5. Sensitivity of EPS-605 Solution to Fe^3+^

**Figure 5:**
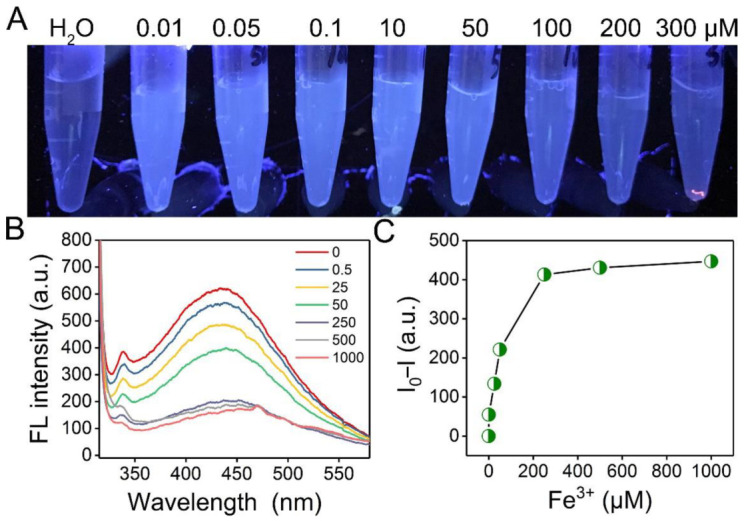
(**A**) Photographs taken under 302 nm UV light of EPS solutions (0.8 mg/mL) in the presence of various concentrations of Fe^3+^. (**B**) Fluorescence emission spectra of EPS-605 (0.8 mg/L) in the presence of various concentrations of Fe^3+^ in an aqueous solution under 302 nm excitation. (**C**) Plots of the changes of fluorescence intensity (I − I_0_) versus Fe^3+^ at different concentrations.

To evaluate the sensitivity of EPS-605 to Fe^3+^, the influence of Fe^3+^ concentrations on the fluorescence property of EPS-605 solutions was detected by fluorescence spectroscopy at an excitation of 302 nm, while the photos of EPS-605 solutions (0.8 mg/mL) treated with different concentrations of Fe^3+^ (0.5, 25, 50, 250, 500, and 1000 μM) were also obtained under UV light irradiation (302 nm) (Figure 5A). As the Fe^3+^ concentration increased, the fluorescence intensity of the EPS-605 solution decreased (Figure 5B). Figure 5C presents the relationship of the changes in fluorescence intensity of EPS-605 in the presence of various concentrations of Fe^3+^ at 302 nm. As the concentration of Fe^3+^ increased, the fluorescence intensity decreased substantially, exhibiting its good sensing ability to Fe^3+^. The relative fluorescence intensity change of EPS-605 was observed to be linearly proportional to the Fe^3+^ concentration in the range from 0.5 to 250 μM (Figure S3), which matched well with the Stern−Volmer equation. In addition, the limit of detection was calculated to be 0.06 μM (at a signal-to-noise ratio of 3).

### 3.6. Emission Mechanism of EPS-605 and Quenching Mechanism in the Presence of Fe^3+^

**Figure 6:**
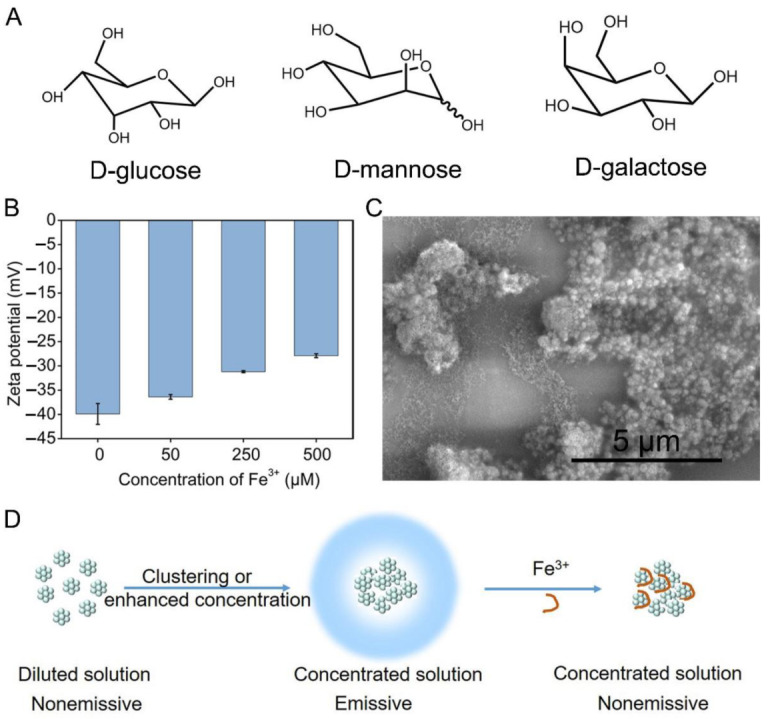
(**A**) Monosaccharide unit structure of EPS-605. (**B**) Zeta potential changes of EPS-605 in the presence of different concentrations of Fe^3+^. (**C**) SEM images of EPS-605 (0.8 mg/mL) in the presence of Fe^3+^ at the concentration of 250 μM (**C**). (**D**) Emission mechanism of EPS-605 and quenching mechanism in the presence of Fe^3+^.

In previous work that studied the intrinsic blue photoluminescence (PL) properties of rice, it was reported that the oxygen clusters formed by the electron-rich oxygen atoms in rice are helpful for the intrinsic PL under UV irradiation [11,19]. Meanwhile, the natural polysaccharide product, chitosan, glucan, dextran, glycogen, glucose, xylose, galactose, and fructose are reported to emit fluorescence in the solid state based on the CTE mechanism [19,28,43,44]. EPS-605 mainly consists of heteropolysaccharides that are composed of mannose, glucose, and galactose (Figure 6A). The intra- and intermolecular O…O interactions of such monosaccharides were the main reason for fluorescence emission in EPS-605. Furthermore, abundant hydrogen bonds formed between the monosaccharides also facilitate O…O interactions and stiffen the molecular conformations [45]. Moreover, modifications for EPS-605, such as acylation, phosphorylation, sulfation, and carboxylation, were also observed in EPS-605 [30], which also contribute to the intrinsic luminescence of EPS-605 [11].

To investigate the fluorescence quenching mechanism of EPS-605 with Fe^3+^, we characterized additional properties of EPS-605, including zeta potential, SEM, and FTIR. The zeta potential of the EPS-605 solution increased from –37.9 to –27.9 mV (Figure 6B). SEM shows that some reticular structures were formed surrounding or on the surface of the EPS-605 nanoparticles (Figure 6C), probably due to the interaction between the carboxyl/hydroxyl groups on the surface of EPS-605 and the introduced Fe^3+^. As shown in the FTIR spectrum (Figure S4), in addition to the typical functional groups of polysaccharides, such as –OH stretching at 3433 cm^−1^, C–H stretching vibration with a peak at 2930 cm^−1^, carboxyl groups (1700–1600 cm^−1^), and C−O at 1068 cm^−1^, a new peak attributed to the Fe–O stretching at around 579 cm^−1^ was found in the EPS-605 sample with Fe^3+^. The results further confirm that the detection of Fe^3+^ is a static fluorescence quenching process.

In summary, as illustrated in Figure 6D, EPS-605 nanoparticles are dispersed in dilute solutions with isolated oxygen atoms owing to the electrostatic repulsion among the negatively charged nanoparticles, thus making them virtually nonluminescent. As the concentration increases or EPS-605 forms aggregations, such as in a film or powder state, the electrostatic charges are screened by the redistributed or overlapped charges. Thus, the molecules can be close to each other, making oxygen atoms and carboxylates closely clustered to emit fluorescence [12]. Considering the CTE property and biosafety of EPS-605, they can also be used in bioimaging, where they can aggregate.

## 4. Conclusions

In summary, the emission behaviors of naturally occurring EPS-605 nanoparticles without conventional chromophores were comprehensively investigated. EPS-605 nanoparticles are practically nonluminescent in dilute solutions but become highly emissive in concentrated solutions, solid powder, and film. The fluorescence and confocal images proved that the fluorescence phenomenon was not affected by pH and the carbon sources. The CTE mechanism can be used to explain the emission behavior of EPS-605. Furthermore, in an aqueous solution, EPS-605 nanoparticles exhibit high sensitivity and selectivity toward Fe^3+^ ions with a detection of limit of 0.06 μM. The complexation of EPS-605 nanoparticles with Fe^3+^ results in the quenching of EPS-605 fluorescence by forming the reticular structure between EPS-605 and Fe^3+^ ions.

## Data Availability

The data presented in this study are available upon request from the corresponding author.

## Supplementary Materials

The following supporting information can be downloaded at: https://www.mdpi.com/article/10.3390/polym14194050/s1: Figure S1: Photographs of EPS-605 aqueous solutions with different concentrations under green-emitting laser irradiation; Figure S2: Photographs of EPS-605 solutions (0.8 mg/mL) taken under 302 nm UV light; Figure S3: Plots of the relationship between ΔI/I and Fe^3+^ concentration; Figure S4: FTIR spectra of EPS-605 in the presence of Fe^3+^.
