# Supplementary material for: Clustering-Triggered Emission of EPS-605 Nanoparticles and Their Application in Biosensing
Source: Polymers (Basel). 2022 Sep 27;14(19):4050. doi: 10.3390/polym14194050 (PMC9571269; doi:10.3390/polym14194050)
Supplement: Supplementary file 1 [file polymers-14-04050-s001.zip › polymers-1922117-supplementary.pdf]

Supplementary Information

Clustering-Triggered Emission of EPS-605 Nanoparticles and Their Application in Biosensing

Chengcheng Li [1], Xiaotong Shi [1] and Xiaodong Zhang [2,*]

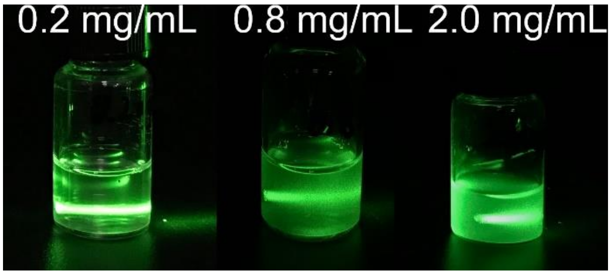

Figure S1. Photographs of EPS-605 aqueous solutions with different concentrations under green-emitting laser irradiation.

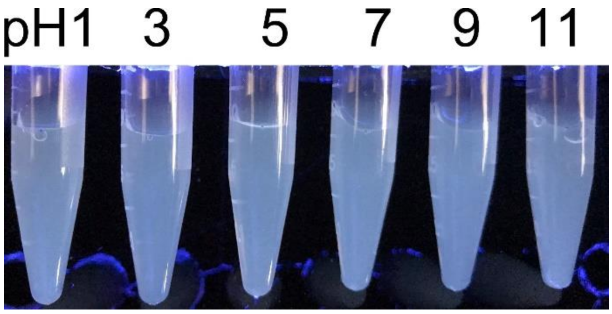

Figure S2. Photographs of EPS-605 solutions (0.8 mg/mL) taken under 302 nm UV light.

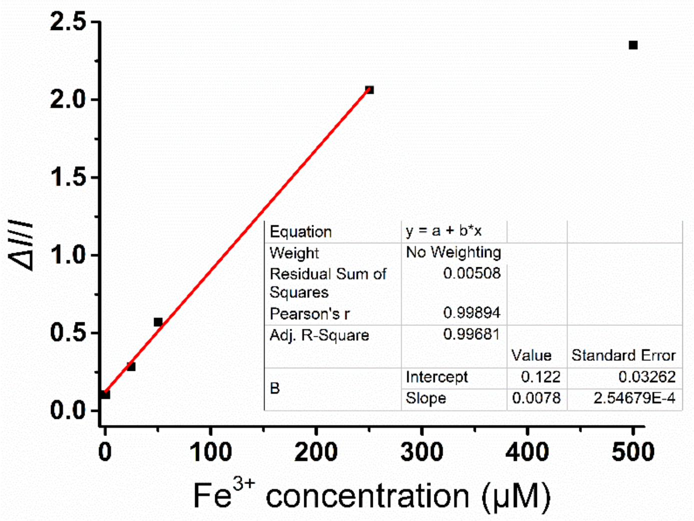

Figure S3. Plots of the relationship between *ΔI*/*I* and Fe^{3+} concentration.

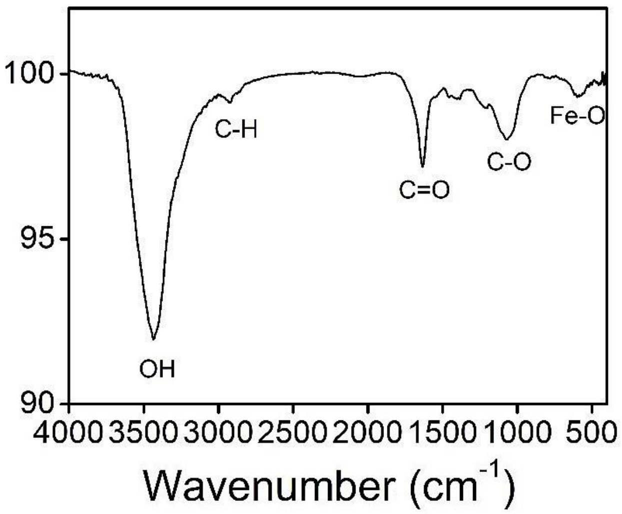

Figure S4. FTIR spectra of EPS-605 in the presence of Fe^{3+}.
